# Investigation of Lethal Concurrent Outbreak of Chlamydiosis and Pigeon Circovirus in a Zoo

**DOI:** 10.3390/ani11061654

**Published:** 2021-06-02

**Authors:** Wei-Tao Chen, Chin-Ann Teng, Cheng-Hsin Shih, Wei-Hsiang Huang, Yi-Fan Jiang, Hui-Wen Chang, Chian-Ren Jeng, Yen-Hsueh Lai, Jun-Cheng Guo, Pao-Jung Wang, Chiu-Hung Cheng, Yen-Chen Chang

**Affiliations:** 1 Graduate Institute of Molecular and Comparative Pathobiology, School of Veterinary Medicine, National Taiwan University, Taipei 10617, Taiwan; b04609022@ntu.edu.tw (W.-T.C.); r07644007@ntu.edu.tw (C.-A.T.); r06644001@ntu.edu.tw (C.-H.S.); whhuang@ntu.edu.tw (W.-H.H.); yfjiang@ntu.edu.tw (Y.-F.J.); huiwenchang@ntu.edu.tw (H.-W.C.); crjeng@ntu.edu.tw (C.-R.J.); 2 Taipei Zoo, Taipei 116016, Taiwan; sux09@zoo.gov.tw (Y.-H.L.); sux08@zoo.gov.tw (J.-C.G.); sux06@zoo.gov.tw (P.-J.W.); dwx37@zoo.gov.tw (C.-H.C.)

**Keywords:** *Chlamydia psittaci*, *Columbiformes*, pigeon circovirus, zoo

## Abstract

**Simple Summary:**

The present study aimed to investigate a lethal outbreak of chlamydiosis and pigeon circovirus (PiCV) infection in a zoo. A retrospective follow-up indicates that the lethal outbreak might be an independent episode. The high prevalence of PiCV positivity in the aviaries suggests that PiCV infection might play a key role in augmenting the lethality of chlamydiosis in birds. Persistently monitoring both pathogens and identifying potential PiCV carriers or transmitters might also help prevent lethal disease outbreaks.

**Abstract:**

During the spring, an outbreak of sudden death involving 58 birds occurred in a zoo. Histopathological examinations revealed variable numbers of intracytoplasmic basophilic microorganisms in the macrophages, hepatocytes, and renal epithelium of most birds, along with occasional botryoid intracytoplasmic inclusion bodies within histiocytes in the bursa of Fabricius. Based on the results of histopathological examinations, immunohistochemical staining, transmission electron microscopy, and polymerase chain reactions, genotype B *Chlamydia psittaci* infection concurrent with pigeon circovirus (PiCV) was diagnosed. A retrospective survey, including two years before the outbreak and the outbreak year, of *C. psittaci* and PiCV infections of dead birds in the aviaries, revealed that the outbreak was an independent episode. The findings of this study indicate that concurrent infection with *C. psittaci* and PiCV might lead to lethal outbreaks of chlamydiosis, particularly *Streptopelia orientalis.* In addition, persistently monitoring both pathogens and identifying potential PiCV carriers or transmitters might also help prevent lethal disease outbreaks.

## 1. Introduction

*Chlamydia psittaci* (*C. psittaci*) is a gram-negative obligate intracellular bacterium that can infect a wide range of hosts [1]. In birds, more than 470 species from at least 30 orders are known to be susceptible to *C. psittaci* infection, among which psittacine birds, pigeons, and mynah birds are most commonly affected [2]. Given that infected birds can intermittently shed pathogens in fecal droppings, dander, and respiratory secretions, bird-to-bird transmission generally occurs via the ingestion of water or feed contaminated by fecal droppings or respiratory secretions or by inhalation of aerosols emitted by affected birds [3]. Infection of *C. psittaci* in birds, also known as avian chlamydiosis or psittacosis, gives rise to a range of non-specific clinical manifestations with various degrees of severity, and in extreme cases, sudden death. The mortality rate varies according to the pathogen strain and infected host species [4]. However, subclinical infections have also been frequently reported in pigeons, older psittacine birds, and poultry [5]. *C. psittaci* can infect humans via inhalation of contaminants or contact with the eyes, resulting in asymptomatic signs, flu-like symptoms, or severe pneumonia. Individuals who are routinely in close contact with birds, such as birdkeepers, poultry workers, and veterinarians, are at a high risk of infection. However, passing or transient contact with feral pigeons results in many (40%) of reported cases in humans [6,7,8].

Based on the outer membrane protein A (*ompA*) gene that encodes the major outer membrane protein and contains several host-determinant and serotype-specific epitopes [9], there are 10 genotypes, designated A to G, E/B, WC, and M56, associated with differences in host preference and virulence. Genotypes B and E are commonly found in pigeons, whereas the genotype A infecting psittacine birds is a common pathogen that can cause respiratory disease in humans [9,10]. Nevertheless, it has been reported that regardless of genotype, *C. psittaci* can jump between species [11,12,13].

In the present study, we report a lethal outbreak of *C. psittaci* in Columbiformes species in the aviaries, which were managed by the same group of keepers in a zoo. To verify the beginning and the end of *C. psittaci* infection, we detected *C. psittaci* antigens in formalin-fixed paraffin-embedded (FFPE) tissue blocks prepared from necropsy cases originating from the same aviaries two years before and after the 3-month outbreak, until the end of the outbreak year. In addition, we also assessed all birds for the presence of pigeon circovirus (PiCV), which is conceivably a risk factor for heightened susceptibility to chlamydiosis in Columbiformes birds and a potential source of infection for other non-Columbiformes species.

## 2. Materials and Methods

### 2.1. Gross and Histopathological Examinations

During the spring, 60 birds [*Aix sponsa* (*n* = 1), *Anas platyrhynchos* (*n* = 1), *Argusianus argus* (*n* = 1), *Caloenas nicobarica* (*n* = 2), Chalcophaps indica (*n* = 1), *Columba pulchricollis* (*n* = 2), *Spilopelia chinensis* (*n* = 2), *Streptopelia orientalis* (*n* = 47), and *Streptopelia tranquebarica* (*n* = 3)] were found dead in the same aviaries, which were housed 96 species of birds, in a zoo and the total mortality rate was approximately 10%. We performed gross examinations of the corpses, from which representative tissues were sampled and fixed in 10% neutral buffered formalin. The fixed tissues were trimmed and embedded in paraffin, following standard protocols. FFPE tissue blocks were sectioned at a width of 4 μm and stained with hematoxylin and eosin (H&E) using a routine staining protocol. Prepared slides were read and diagnosed by veterinary pathologists at the Graduate Institute of Molecular and Comparative Pathobiology, School of Veterinary Medicine, National Taiwan University, Taiwan. 

### 2.2. DNA Extraction from Fresh Tissues and Formalin-Fixed Paraffin-Embedded Tissue Blocks

A total of 44 and 69 avian tissue samples were collected two years before the outbreak and the year of the outbreak, respectively, and the corresponding bird species and their numbers are listed in Table 1. The examined samples included 62 FFPE blocks collected from the two years before the outbreak and after it until the end of the year, and 51 fresh liver tissues collected during the outbreak. In addition, all tissue blocks were composed of multiple organs, including the liver, spleen, kidney, intestine, or bursa of Fabricius. For the preparation of FFPE blocks, tissue slices at a thickness of 4 μm were deparaffinized twice by mixing with 1 mL of non-xylene (Muto Chemical, Tokyo, Japan), vortexed, and then centrifuged at 1000× *g* for 5 min to remove the paraffin-containing supernatant. Thereafter, the tissues were washed twice by resuspending in 1 mL of 99% ethanol (Sigma-Aldrich, St. Louis, MO, USA) and pelleted by centrifugation at 1000× *g* for 5 min. After discarding the supernatant, the pelleted tissue was air-dried to remove ethanol and subsequently used for DNA extraction. DNA was extracted from FFPE tissues and fresh liver using a DNeasy Blood and Tissue Kit (Qiagen, Hilden, Germany) according to the manufacturer’s instructions, and DNA samples were stored at −20 °C for subsequent use.

### 2.3. Polymerase Chain Reaction Analyses of GAPDH and the 16S Ribosomal RNA (rRNA) and ompA Gene of Chlamydia psittaci 

All prepared DNA samples were analyzed for *GAPDH*, a housekeeping gene, to assess DNA quality, as previously described [14], and screened using primers targeting the 16S rRNA gene of *C. psittaci* [15], with subsequent nucleotide sequencing confirmation performed by Tri-IBiotech Inc., Taipei, Taiwan. Genotyping of all *C. psittaci*-positive samples from fresh tissue was performed by amplifying and sequencing the partial *ompA* gene with a length of 1041 base pairs (bp) [16]. Reaction mixtures comprised 1 μL of DNA sample, 10 μL of AmaR OnePCR^TM^ reagent (GeneDirectX, Taoyuan, Taiwan), 1 μL of each primer, and 7 μL of PCR-grade water. Amplification of 16S rRNA and *GAPDH* was performed using the following program: initial denaturation at 94 °C for 3 min; followed by 35 cycles of denaturation at 94 °C for 30 s, annealing at 60 °C for 30 s, and extension at 72 °C for 30 s; with a final extension at 72 °C for 5 min. The PCR conditions used for amplification of the *ompA* gene were similar to those described for 16S rRNA and *GAPDH*, with the exception that we used an annealing temperature of 54 °C and an extension time of 80 s. The PCR products thus obtained were separated by gel electrophoresis using 2% agarose gels, and the amplicons of requisite target sizes were purified using a QIAquick Gel Extraction Kit (Qiagen, Valencia, CA, USA) and subjected to nucleotide sequencing. 

### 2.4. Polymerase Chain Reaction Detection of Pigeon Circovirus 

All DNA samples were subsequently screened for the PiCV capsid protein-encoding C1 gene, using previously published primers [17], and the PCR conditions were performed using the following program: initial denaturation at 94 °C for 3 min; followed by 35 cycles of denaturation at 94 °C for 30 s, annealing at 60 °C for 30 s, and extension at 72 °C for 30 s; with a final extension at 72 °C for 5 min. Following gel electrophoresis confirmation, the purified amplicons were sent for nucleotide sequencing. 

### 2.5. Transmission Electron Microscopy (TEM)

Formalin-fixed liver tissue was cut into 1-mm^3^ cubes, consecutively fixed in 2.5% glutaraldehyde overnight, and post-fixed in 1% osmium tetroxide for 90 min. The cubes were processed through standard ethanol dehydration and embedded in Spurr’s resin. Ultrathin sections (70 nm in width) were cut using a Leica EM UC7 ultramicrotome (Leica Microsystems, Wetzlar, Germany) and stained with 10% methanolic uranyl acetate and Reynold’s lead citrate. Images were acquired using a Tecnai G2 F20 S-TWIN transmission electron microscope (FEI Company, Hillsboro, OR, USA) operated at 120 kV.

### 2.6. Immunohistochemical Staining of C. psittaci

The FFPE tissue blocks of the cases with target-sized amplicons were sliced at a width of 4 μm and placed on Immuno Coat Slides (Muto Pure Chemicals, Tokyo, Japan). The tissue slides were deparaffinized with xylene and rehydrated in a gradient series of ethanol solutions using a standard protocol. Antigens were retrieved by boiling in 5% Trilogy (Cell Marque, Rocklin, CA, USA) using Montage Opus^TM^ (Diagnostic Biosystems, Pleasanton, CA, USA) for 20 min. Thereafter, the tissues were blocked with 5% goat normal serum (Dako, Glostrup, Denmark) at room temperature (RT) for 1 h and then interacted with a monoclonal *Chlamydia trachomatis* antibody (Fitzgerald Industries, Concord, MA, USA) (showing cross-reactivity with *C. psittaci*) diluted 200-fold in Primary Antibody Diluent (Tris Green; ScyTek Laboratories, Logan, UT, USA), at RT for 1 h. Endogenous peroxidase was inactivated by 1% hydrogen peroxide for 10 min at RT, and following three washes with TBST, BioGenex^®^ Super Enhancer ™ (BioGenex, San Ramon, CA, USA) was applied at RT for 30 min. Development was performed using Dako EnVision+ System-HRP 3,3-diaminobenzidine (Dako) with hematoxylin counterstaining. The brown spots within the susceptible cells were interpreted as a positive signal indicating the presence of *Chlamydia*.

### 2.7. Sequence Analysis 

Multiple sequence alignments of the 16S rRNA and *ompA* genes of *C. psittaci* and the *cap* gene of PiCV were performed using MEGA X software based on the Clustal W method [18]. MEGA X was also used to construct a phylogenetic tree based on the maximum likelihood method and the Kimura 2-parameter model, with bootstrapping for 1000 replications.

## 3. Results

### 3.1. Gross Examination

Within three months, sixty birds were found dead in the zoo, most of which showed moderate to poor nutritional conditions, characterized by a reduced pectoral muscle volume without evidence of obvious trauma. Necropsy in most of the birds revealed similar gross lesions, including the thickening of air sacs with fibrinous substances and the adherence of the pericardium and serosa of the liver, spleen, and gastrointestinal tract to variable amounts of similar fibrinous substances. In addition, we detected moderate to marked hepatomegaly, splenomegaly, and renomegaly. 

### 3.2. Histopathological Examination

**Figure 1:**
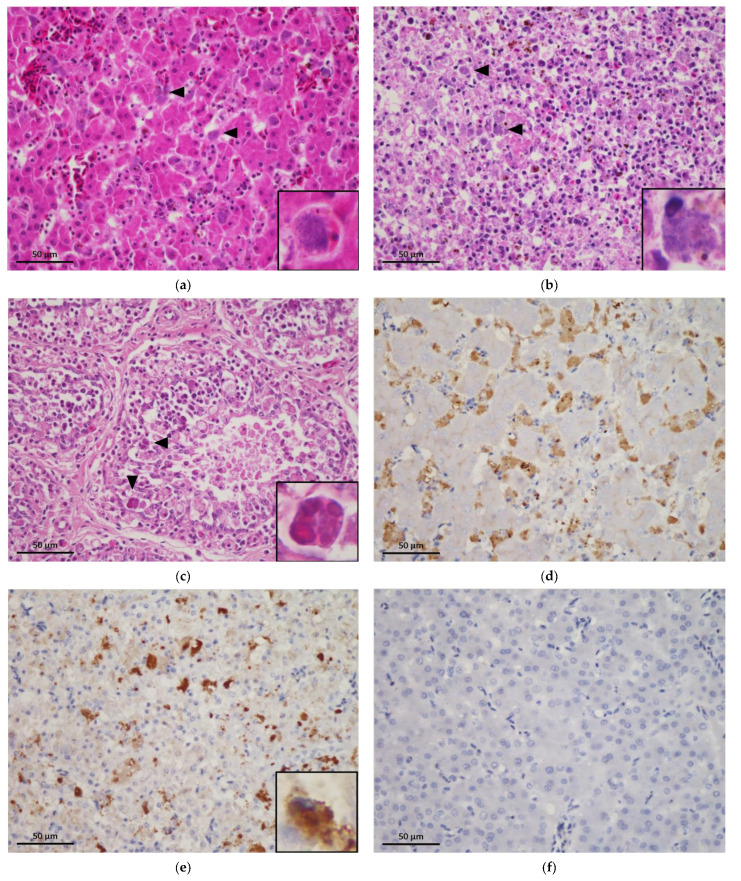
The result of histopathological examination and immunohistochemical (IHC) staining. (**a**) In the liver, there are various numbers of intracytoplasmic basophilic punctate organisms in the hepatocytes and macrophages (arrowheads); H&E stain, ×400. (**b**) Intracytoplasmic basophilic punctate organisms distributed in splenic macrophages (arrowheads); H&E stain, ×400. (**c**) Large botryoid inclusion bodies (indicated by the arrowheads) within the cytoplasm of macrophages in the bursa of Fabricius with lymphoid depletion; H&E stain, ×400. (**d**,**e**) Strong *Chlamydia*-positive signals detected within the cytoplasm of macrophages in the liver and spleen, respectively; IHC stain, ×400. (**f**) A liver section of a PCR-negative case subjected to IHC staining; IHC stain, ×400. The insets in panels (**a**) to (**e**) show infected cells under ×1000 magnification.

Histopathological examination of 53 of the 60 birds (88.3%) collected during the outbreak revealed histiocytic inflammation in multiple organs (Table 2), with diffuse, severe histiocytic hepatitis, histiocytic splenitis, and fibrinous histiocytic airsacculitis; in particular, numerous intracytoplasmic, basophilic, and punctate microorganisms were predominantly present in the histiocytes of the spleen and liver Kupffer cells (Figure 1a,b). Interstitial nephritis with massive renal tubular necrosis and histiocytic meningitis was observed in 17/60 (28.3%) and 8/60 (13.3%) cases, respectively. These microorganisms, which stained dark blue by Giemsa staining and were acid-fast stain-negative, were also detected in histiocytes in the air sac, pericardium, meninges, serosa of the gastrointestinal tract, and renal tubular epithelial cells. Furthermore, the bursa of Fabricius in three *Streptopelia orientalis* showed diffuse lymphoid depletion, with large botryoid intracytoplasmic inclusion bodies observed in the macrophages (Figure 1c). No detectable PiCV-associated histopathological lesion was noted in any non-Columbiformes birds or birds collected before and after the outbreak period.

### 3.3. Transmission Electron Microscopy of C. psittaci

**Figure 2:**
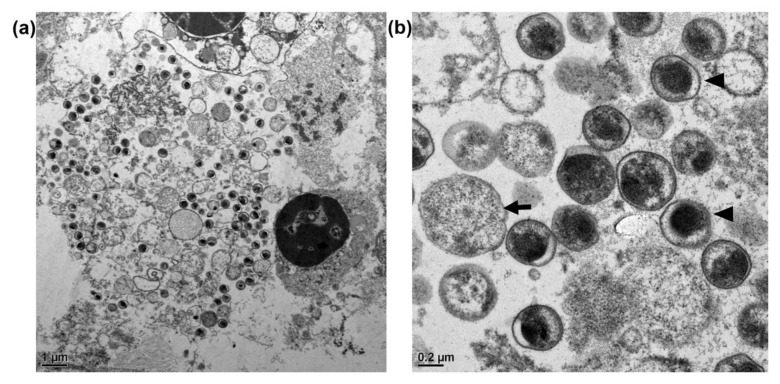
Transmission electron microscopy images of *Chlamydia psittaci*. (**a**) The cytoplasm of infected cells is occupied by numerous variably sized vesicles of different electron densities. (**b**) The elementary bodies of *C. psittaci* are small electron-dense spherical bodies, ranging in diameter from 0.4 to 0.5 μm, with distinct periplasmic electron-transparent spaces (arrowheads). The reticulate bodies (arrow), ranging in diameter from 0.5 to 1.5 μm, are characterized by large amounts of granular cytoplasm.

Within the cytoplasm of infected cells, we detected numerous internalized spherical organisms that were morphologically identical to the elementary bodies (EBs), reticulate bodies, and intermediate bodies of *C. psittaci*. The majority of spherical organisms were EBs of 0.4 to 0.6 μm in diameter, with an electron-dense eccentric nucleus and occasional periplasmic spaces (Figure 2). 

### 3.4. Sequence Analysis of C. psittaci 

**Figure 3:**
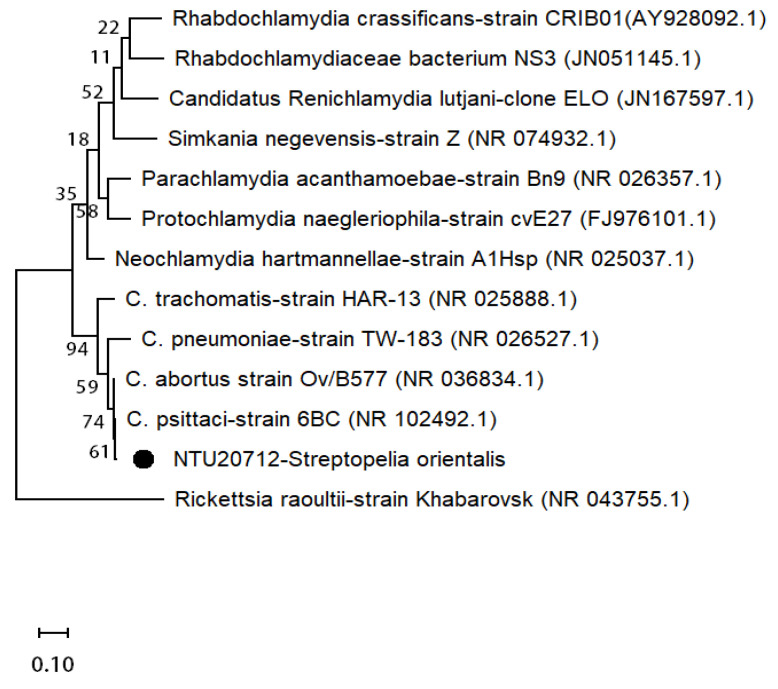
Phylogenetic analysis of *Chlamydia psittaci* based on sequences of the 16S rRNA gene. The black dot indicates the *C. psittaci* strain detected in the present outbreak. The GenBank accession numbers are shown in parentheses.

The percentage PCR-positive detection of 16S rRNA for specimens collected in the prior two years and the outbreak year was 25.0% (11/44) and 84.3% (59/69), respectively. Nucleotide sequencing of the target-sized amplicons revealed a total of 58 cases positive for *C. psittaci* in the outbreak year (Table 2 and Figure 3), whereas, in contrast, none of the cases in the prior two years were identified as *C. psittaci* positive. The *C. psittaci* sequences (GenBank accession number MW301282-MW301338) were all obtained from necropsied birds during the outbreak period and found to have very similar identities of 99.5% to 100%. Interestingly, other 12 16S rRNA-positive cases (11 cases in the prior two years and 1 case in the outbreak year) were all identified as non-Chlamydiaceae Chlamydiales. 

**Figure 4:**
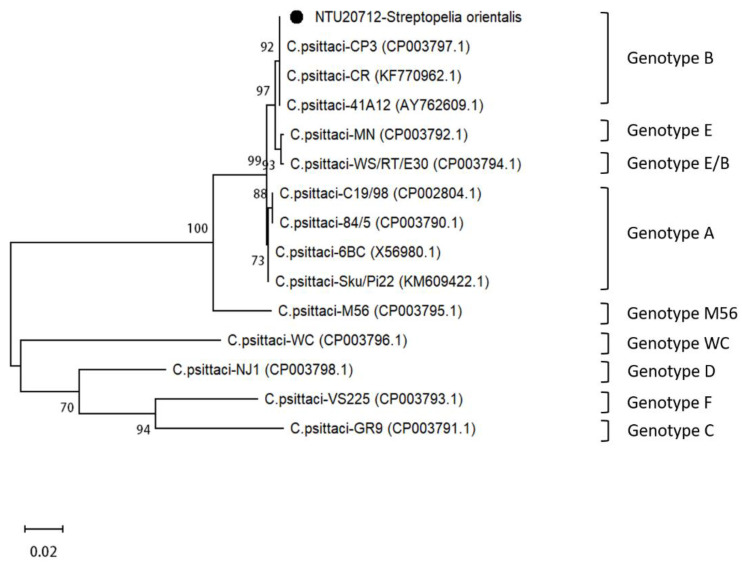
Phylogenetic analysis of *Chlamydia psittaci* based on sequences of the *ompA* gene. The black dot indicates the *C. psittaci* strain detected in the present outbreak. The GenBank accession numbers are shown in parentheses.

After comparing the *ompA* gene in *C. psittaci*-positive cases, the nucleotide identity ranged from 99.9 to 100%. Thus, a representative sample (NTU20712) derived from a *Streptopelia orientalis* specimen was used for genotyping *C. psittaci*, targeting the *ompA* gene, which revealed 100% nucleotide identity with the genotype B CP3 strain (GenBank accession number AF269265.1) (Figure 4).

### 3.5. Immunohistochemical Staining of C. psittaci

The 16S rRNA PCR-positive samples (*n* = 70), comprising *C. psittaci*-positive (*n* = 58) and NCC-positive (*n* = 12) cases, were analyzed using immunohistochemical staining to determine the association between *C. psittaci* and observed lesions. The species and number of positive cases are listed in Table 2. Among the 58 *C. psittaci*-PCR-positive cases, 53 showed significant immunoreactivity in bacterial clusters, and positive signals were also detected in the cytoplasm of macrophages within the tissue lesions (Figure 1d,e). Furthermore, positive signals were also detected in the cytoplasm of hepatocytes and renal epithelial cells. In contrast, we detected no positive signal in the NCC PCR-positive tissues.

### 3.6. Sequence Analysis of Pigeon Circovirus

**Figure 5:**
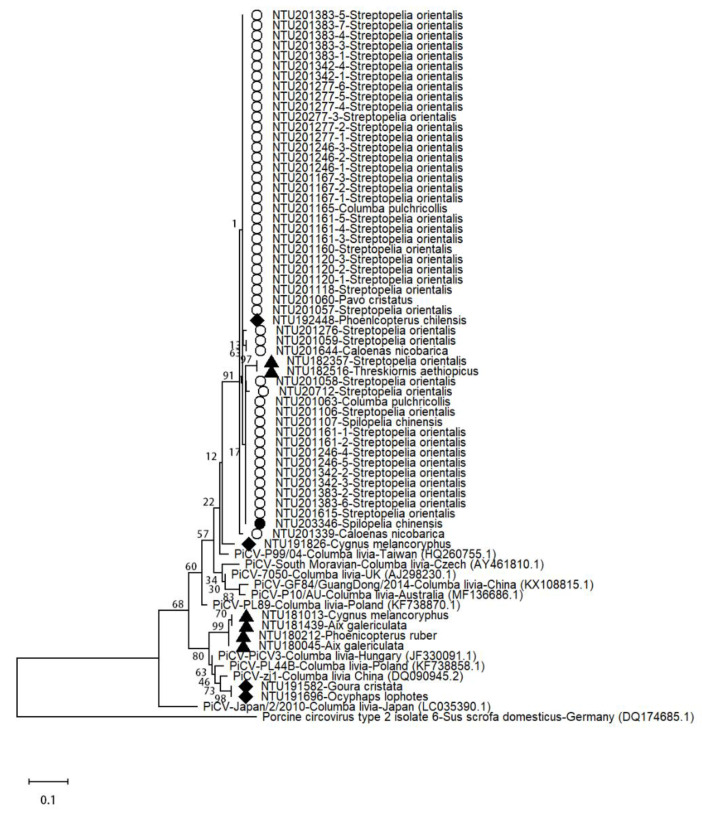
Phylogenetic analysis of pigeon circovirus based on partial *cap* gene sequences. The information on viral isolates, including first reported animal species and location and GenBank accession number, is shown. The cases labeled with the same indicator are collected in the same year. ○, cases occurred in the outbreak; ●, cases occurred after the outbreak; ▲and ♦, cases occurred before the outbreak; PiCV, pigeon circovirus.

The PiCV nucleotides were detected in 5 orders, including Anseriformes, Columbiformes, Galliformes, Pelecaniformes, and Phoenicopteriformes and in 12 species [*Aix galericulata* (*n* = 2), *Caloenas nicobarica* (*n* = 2), *Columba pulchricollis* (*n* = 2), *Cygnus melancoryphus* (*n* = 2), *Goura cristata* (*n* = 1), *Ocyphaps lophotes* (*n* = 1), *Pavo cristatus* (*n* = 1), *Phoenicopterus chilensis* (*n* = 1), *Phoenicopterus ruber* (*n* = 1), *Spilopelia chinensis* (*n* = 2), *Steptopelia orientalis* (*n* = 43), and *Threskiornis aethiopicus* (*n* = 1)] (Table 3). The nucleotide sequence identities ranged from 80% to 100% (GenBank accession numbers MW322587-MW322648), and the phylogenetic results are shown in Figure 5. 

In the outbreak, 81% (47/58) of the *C. psittaci*-positive birds were also positive for PiCV, namely *Caloenas nicobarica* (*n* = 2), *Columba pulchricollis* (*n* = 2), *Spilopelia chinensis* (*n* = 2), and *Streptopelia orientalis* (*n* = 41), with sequence identities ranging from 97.8% to 100%. Compared to other reference strains, the detected virus was found to have 91.2% to 93.2% nucleotide identity with the Taiwan P99/04 strain and high sequence identity with strains PiCV/P10/AUS (GenBank accession number MF136686.1), South Moravian 1 (GenBank accession number AY461810.1), PL89 (GenBank accession number KF738870.1), and 7050 (GenBank accession number AJ298230.1), ranging from 89.1% to 92.5%. Corresponding comparisons of the amino acid sequences revealed sequence identities between 97.9% and 100% among the detected isolates and with the Taiwan P99/04 strain and identities ranging from 95.8% to 100% with the other previously published strains.

The nucleotide sequences of the isolates before the outbreak were identical to those of the PiCV3 strain (94–95%) (GenBank accession number JF330091.1) or zj1 strain (97%) (GenBank accession number DQ090945.2), suggesting that several isolates were circulating in the aviaries. The nucleotide sequence was not significantly different between Columbiformes and non-Columbiformes birds, but the isolates collected from the same year seemed to share higher nucleotide identity, such as NTU182357 and NTU18-2516; NTU180045, NTU181013, NTU180212, and NTU181439; NTU191582 and NTU191696; and all cases in the outbreak year (cases labeled with circles in Figure 5). 

## 4. Discussion

*C. psittaci* is an important zoonotic pathogen that could infect a wide range of hosts. In the current study, we confirmed a devastating outbreak of avian chlamydiosis, claiming 58 birds in the zoo, based on histopathological examinations, TEM observations, immunohistochemical staining, and PCR analyses. A retrospective study of *C. psittaci* infections in the aviaries revealed no positive cases in PCR analyses targeting the *C. psittaci* 16S rRNA gene in tissue sections prepared during the two years before the outbreak, thereby indicating an absence of latent infection in the aviaries and indicating that the lethal outbreak is likely to have been an independent episode. Phylogenetic analyses revealed that the causal *C. psittaci* is closely related to the genotype B CP3 strain, which is typically identified in pigeons [9,19]. Our observations thus indicate that, in addition to pigeons [20], *Aix galericulata*, *Caloenas nicobarica*, *Columba pulchricollis*, *Spilopelia chinensis*, *Streptopelia orientalis*, and *Streptopelia tranquebarica* are also susceptible to genotype B *C. psittaci*. 

In the present case, it is notable that compared with Columbiformes birds, and particularly *Streptopelia orientalis*, species in other avian orders, including Gruiformes, Anseriformes, and Galliformes, in the same aviaries were less affected. Of note in respect, there are large groups of resident feral pigeons within the grounds of the zoo, and the netting cannot completely prevent contact or environmental contamination from the droppings and danger of wild birds. Feral pigeons, which are commonly found in urban areas and are frequently in close contact with humans and other animals, are considered among the common reservoirs of zoonotic diseases [21], and it has been reported that the percentage PCR-positive detection of *C. psittaci* in these pigeons ranges from 16% to 36.7% in Beijing, China, 11.1% to 100% (average 22.9%) in Japan, and 3.4% to 95.6% in Europe [8,20]. Comparatively, in Taiwan, Liu et al. have indicated that the prevalence of *C. psittaci* infection in racing pigeons and other Columbidae and wild birds from wildlife refuges is 10.1% and 2.2%, respectively [19]. The possibility of transmission of *C. psittaci* from wild asymptomatic pigeons or Gruiformes, Anseriformes, and Galliformes in the same aviaries could not be completely excluded in this case. 

Several genes have been reported as potential virulence factors of *C. psittaci* based on comparisons of the sequences of virulent and attenuated strains [3,9,22]. Interestingly, an identical amino acid substitution (S497G) previously described in the virulent 6BC strain was detected in our study, as well as the genotype B CP3 strain, which has been considered a low-virulence strain in specific-pathogen-free chickens [23]. This particular substitution was reported as a potential virulence factor in an investigation of a previous atypical lethal outbreak in Columbiformes birds [3]. Since the role of the amino acid substitution in the *C. psittaci pkn5* gene has yet to be investigated in Columbiformes species, particularly *Streptopelia orientalis*, further investigation to study the virulence factors of *C. psittaci* in Columbiformes species is needed. 

Another important finding of the present study was the co-infection of deceased birds with *C. psittaci* and the PiCV. Previous studies have indicated that although PiCV infection may cause disease without evident symptoms, concurrent secondary infections usually result in an increased mortality rate [2,24]. Similar to *C. psittaci*, the routes of transmission are primarily via inhalation or ingestion of material contaminated with feces or feather dust from affected individuals, and vertical transmission may also occur [25]. The virus typically propagates in proliferating cells and immune cells, resulting in lymphoid atrophy and lymphocyte depletion, particularly in young birds up to 6 months of age, and it is accordingly considered an immunosuppressive factor for pigeons that might contribute to superinfection with bacteria or other viruses. Indeed, Stenzel et al. (2014) demonstrated that the number of birds co-infected with PiCV and *C. psittaci* was two to three times higher than that of birds infected with *C. psittaci* alone [2]. The high prevalence of concurrent PiCV and *C. psittaci* infection in the deceased Columbiformes in the present outbreak suggests that PiCV might have played an important role in potentiating the most recent outbreak of *C. psittaci* infection or pathogenicity. 

To date, PiCV has only been reported in three Columbiformes birds, including *Columba livia*, *Streptopelia senegalensis* [26], and *Streptopelia decaocto* [27]. Based on the intracytoplasmic inclusions and prominent lymphoid depletion in the bursa of Fabricius in three PiCV-positive cases, we report PiCV infection and PiCV-associated lesions in *Streptopelia orientalis* for the first time. Interestingly, the PiCV *cap* gene was also detected by PCR in the other 16 species across seven orders in the present study (Table 3), but no PiCV-related histopathological lesions were observed in these species. These results suggest that *Streptopelia orientalis* might be more vulnerable to PiCV infection, which might lead to immunosuppression in this species and enhance the pathogenicity of other infections, such as lethal chlamydiosis. The lack of obvious lesions in PiCV-positive Columbiformes and non-Columbiformes birds suggests that these birds might be carriers or potential transmitters of PiCV infection. The frequent detection of PiCV in birds in the aviaries over the period of this survey (Figure 5) also indicates that PiCV was widely circulating in the aviaries. Identifying potential PiCV carriers or transmitters should help prevent lethal disease outbreaks. For phylogenetic analysis of PiCV, full-length *C1* gene sequencing or whole-genome sequencing should be performed. Unfortunately, due to the limitation of the DNA quality of samples from FFPE tissue blocks, primer sets amplifying short amplicons of conserved areas instead of the *C1* gene were used to detect PiCV, which might explain the high nucleotide identity among isolates.

## 5. Conclusions

The present study is the first to report a lethal outbreak of chlamydiosis among Columbiformes species in a zoo; however, the origin of the causal bacterium *C. psittaci* remains to be determined. The high PiCV-positive rate detected in this outbreak indicates that PiCV infection might play a key role in augmenting disease progression, and other PiCV-positive species might be undescribed hosts; however, this requires further confirmation. Based on our observations, we speculate that Columbiformes birds other than pigeons, particularly *Streptopelia orientalis*, might serve as sentinels that could be used to forecast the spread of avian chlamydiosis, given their apparent susceptibility to *C. psittaci* infection and their habitats and feeding habits similar to those of pigeons. Although no further deceased birds from the aviaries tested positive for *C. psittaci*, this pathogen could cause asymptotic infection in a wide range of animals that were initially introduced into the environment. Routine monitoring for disease in animals and humans will be essential to assess the risk to public health and to control or prevent chlamydiosis. In addition, persistent monitoring and identification of potential PiCV carriers or transmitters may also help prevent lethal disease outbreaks.

## Figures and Tables

**Table 1:** The bird species and numbers of the cases collected in the present study.

Order	Species	Sample Number	
		2 Years before the Outbreak	The Outbreak Year
Anseriformes	*Aix galericulata*	2	0
	*Aix sponsa*	2	2
	*Anas platyrhynchos*	4	1
	*Cygnus melancoryphus*	6	0
Columbiformes	*Caloenas nicobarica*	1	3
	*Chalcophaps indica*	0	1
	*Columba pulchricollis*	1	2
	*Ducula bicolor*	2	0
	*Goura cristata*	1	0
	*Ocyphaps lophotes*	3	0
	*Spilopelia chinensis*	0	2
	*Streptopelia orientalis*	1	47
	*Streptopelia tranquebarica*	2	3
Galliformes	*Argusianus argus*	0	1
	*Lophura nycthemera*	0	1
	*Pavo cristatus*	0	1
Gruiformes	*Balearica pavonina*	1	0
	*Balearica regulorum*	1	0
	*Gallinula chloropus*	1	0
Musophagiformes	*Musophaga violacea*	0	1
Pelecaniformes	*Eudocimus ruber*	2	1
	*Pelecanus onocrotalus*	1	0
	*Pelecanus rufescens*	4	0
	*Threskiornis aethiopicus*	2	0
Phoenicopteriformes	*Phoenicopterus chilensis*	6	2
	*Phoenicopterus ruber*	1	1

**Table 2:** A summary of the number of positive cases of *Chlamydia psittaci* in the outbreak year based on different detection methods.

Time	Order	Species	*C. psittaci*			*C. psittaci*-Positive Cases
			PCR	H&E	IHC	
Before outbreak	Galliformes	*Pavo cristatus*	0/1	0/1	NA	0/1
	Phoenicopteriformes	*Phoenicopterus chilensis*	0/2	0/2	NA	0/2
	Phoenicopteriformes	*Phoenicopterus ruber*	0/1	0/1	NA	0/1
Outbreak period	Anseriformes	*Aix sponsa*	1/1	0/1	0/1	1/1
	Anseriformes	*Anas platyrhynchos*	1/1	0/1	0/1	1/1
	Columbiformes	*Caloenas nicobarica*	2/2	1/2	1/2	2/2
	Columbiformes	*Chalcophaps indica*	0/1	0/1	NA	0/1
	Columbiformes	*Columba pulchricollis*	2/2	2/2	2/2	2/2
	Columbiformes	*Spilopelia chinensis*	2/2	2/2	2/2	2/2
	Columbiformes	*Steptopelia orientalis*	46/47	45/47	45/47	46/47
	Columbiformes	*Streptopelia tranquebarica*	3/3	3/3	3/3	3/3
	Galliformes	*Argusianus argus*	1/1	0/1	0/1	1/1
After outbreak	Anseriformes	*Aix sponsa*	0/1	0/1	NA	0/1
	Columbiformes	*Caloenas nicobarica*	0/1	0/1	NA	0/1
	Galliformes	*Lophura nycthemera*	0/1	0/1	NA	0/1
	Musophagiformes	*Musophaga violacea*	0/1	0/1	NA	0/1
	Pelecaniformes	*Eudocimus ruber*	0/1	0/1	NA	0/1

The former and latter numbers denote the number of positive and total analyzed cases, respectively. H&E, hematoxylin and eosin staining; IHC, immunohistochemical staining; NA, not analyzed; PCR, polymerase chain reaction.

**Table 3:** The summary of PiCV-positive cases in the present study.

Order	Species	PiCV Positive
Anseriformes	*Aix galericulata*	2/2
Anseriformes	*Cygnus melancoryphus*	2/6
Columbiformes	*Caloenas nicobarica*	2/4
Columbiformes	*Columba pulchricollis*	2/3
Columbiformes	*Goura cristata*	1/1
Columbiformes	*Ocyphaps lophotes*	1/3
Columbiformes	*Spilopelia chinensis*	2/2
Columbiformes	*Steptopelia orientalis*	43/48
Galliformes	*Pavo cristatus*	1/1
Pelecaniformes	*Threskiornis aethiopicus*	1/2
Phoenicopteriformes	*Phoenicopterus chilensis*	1/8
Phoenicopteriformes	*Phoenicopterus ruber*	1/2

The former and latter numbers denote the number of positive and total analyzed cases, respectively.

## Data Availability

The data presented in this study are available on request from the corresponding author. The data are not publicly available due to the privacy policy of the zoo.
